# Effects of Stir-Frying and Heat–Moisture Treatment on the Physicochemical Quality of Glutinous Rice Flour for Making Taopian, a Traditional Chinese Pastry

**DOI:** 10.3390/foods13132069

**Published:** 2024-06-28

**Authors:** Qiuping Xie, Shanshan Wu, Shiyu Lai, Fayin Ye

**Affiliations:** 1 College of Food Science, Southwest University, Chongqing 400715, China; xqp0059@email.swu.edu.cn (Q.X.); lemonade7533@gmail.com (S.W.); lsy1029@email.swu.edu.cn (S.L.); 2 Chongqing Key Laboratory of Speciality Food Co-Built by Sichuan and Chongqing, Chongqing 400715, China

**Keywords:** heat–moisture treatment, glutinous rice flour, pastry, physicochemical quality

## Abstract

Taopian is a traditional Chinese pastry made from cooked glutinous rice flour. The effects of heat–moisture treatment (110 °C, 4 h; moisture contents 12–36%, *w*/*w*) on the preparation of cooked glutinous rice flour and taopian made from it were compared with the traditional method of stir-frying (180 °C, 30 s). The color of heat–moisture-treated (HMT) flours was darker. HMT flours exhibited a larger mean particle size (89.5–124 μm) and a greater relative crystallinity of starch (23.08–42.92%) and mass fractal dimension (1.77–2.28). The flours exhibited water activity in the range of 0.589–0.631. Although the oil-binding capacity of HMT flours was largely comparable to that of stir-fried flours, HMT flours exhibited a lower water absorption index. Accordingly, the taopian produced with HMT flours exhibited a lower brightness, accompanied by a stronger reddening and yellowing. In addition, more firmly bound water was observed in the taopian produced with HMT flour. The taopian made with HMT flour with a moisture content of 24% exhibited moderate hardness, adhesiveness and cohesiveness and received the highest score for overall acceptability (6.80). These results may be helpful to improve the quality of taopian by applying heat–moisture treatment in the preparation of cooked glutinous rice flour.

## 1. Introduction

Glutinous rice has a unique application in various foods due to its amylopectin-rich nature, i.e., more than 98% total starch. Prior to food preparation, glutinous rice paddy is dehusked and milled into glutinous rice flour (GRF). GRF had been widely used in preparing traditional and novel foods such as glutinous rice dumplings (such as tang-yuan), rice puddings (such as mi-gao and nian-gao), desserts, rice cakes (such as fagao and mochi) [1], baked rice crackers and puffed snacks (such as yukwa). It is also applied in meat products as a nonmeat ingredient, which plays an important role in the formation of the textural qualities of meat products. Some researchers reported that beef patties with GRF increased in fat and moisture retentions during cooking and were juicier and tenderer than the control [2]. Ground pork patties with 3% of GRF showed higher cooking yield and better quality [3].

Taopian is a well-known Chinese pastry mainly made of GRF. It can at least date back to the Qing Dynasty (from AD 1644 to 1911) and originates from the provinces in the upper and middle reaches of the Yangtze River such as Sichuan, Chongqing and Hubei. The most famous taopian is produced in Hechuan District and Yunyang County in Chongqing. The traditional process for the preparation of taopian involves: (1) pretreatment of glutinous rice that includes washing the glutinous rice with fresh water, draining the water, and transferring into an iron wok for stir-frying; (2) milling of the pretreated glutinous rice into cooked GRF; (3) mixing the cooked GRF with icing sugar, maltose syrup, lard oil, a small amount of water and other ingredients; (4) molding the mixture into a cuboid shape; (5) incubating the molded mixture with a water bath for a period of time; and (6) the molded mixture being cut into slice shape. Taopian is widely loved by consumers because of its unique slice shape and soft and chewy texture.

For glutinous rice-based foods, the physiochemical properties and texture attributes are largely reliant on changes in the starch during processing. Researchers have claimed that the palatability of GRF-containing food is affected by milling methods and it could be well-modulated by the pretreatment of glutinous rice before milling. GRF has been made by different milling methods such as wet milling, dry milling and semidry milling. Results have shown that the milling methods could affect the content of components and the physicochemical properties of the resulting GRF [4]. Among them, wet milling is a traditional way to make GRF with a lower degree of starch damage, alpha-amylase activity, and ash, which is suitable for the formulating traditional glutinous rice-based foods [5]. Except for the milling methods, previous studies have proven that the pretreatment has a positive impact on the properties of GRF and its products. Li et al. [6] reported that annealed GRF was more suitable for preparing fast-frozen dumplings compared with the native GRF. Lin et al. [7] found that 60 min of hot-air pretreatment to the highest extent maintained the integrity of the starch granules of semi-dry milled GRF. Wang et al. [8] explored the effect of germination treatment on the properties of GRF and the authors observed that 48 h of germination led to a marked decrease in the peak and final viscosities of the flours and lowered their tendency to retrogradation.

Among various modification methods, heat–moisture treatment is a green method to modify raw starchy food materials and improve the qualities of the final products. Heat–moisture treatment involves treating the materials under hydrothermal conditions with a low moisture content (20–35%) at high temperature (80–140 °C) for a specific duration (20 min–16 h) [9]. Kurinami and Sugimoto [10] reported that heat–moisture treatment of glutinous rice led to an increase in hardness of mochi-kiji. Thuengtung et al. [11] found that the heat–moisture treatment on raw paddy rice slowed down the digestion rate of starch in cooked rice. Thus, the present study was an attempt to investigate the physicochemical differences of cooked GRFs made of traditional stir-fried glutinous rice and heat–moisture-treated glutinous rice. Meanwhile, the qualities of taopian prepared with these cooked GRFs were determined and compared. The obtained results provide a reference for regulating the physicochemical properties of GRF and the qualities of the related GRF-based products, such as taopian.

## 2. Materials and Methods

### 2.1. Materials

Glutinous rice (harvested in October 2022), which contained 9.5% (dry basis) protein, 2.5% (dry basis) fat, 71.9% (dry basis) carbohydrate and 12.5% moisture content, was provided by Heilongjiang Cang Natural Ecological Agriculture Co., Ltd. (Harbin, China). Maltose syrup (70%, *w*/*w*) was purchased from Chunyuan Food Co., Ltd. (Dezhou, China). Edible lard oil was from Lichen Grease Co., Ltd. (Weifang, China). Icing sugar was supplied by Qiaosao Food Co., Ltd. (Yichun, China).

### 2.2. Preparation of Cooked GRF and Taopian

#### 2.2.1. Preparation of Cooked GRF

The control sample was prepared by the traditional method: 300 g of fine river sand (100-mesh) was poured into an iron wok with a diameter of 340 mm and depth of 110 mm and preheated on an electromagnetic oven (RH35, Zhongshan Guanwei Environmental Protection Technology Co., Ltd., Zhongshan, China). The temperature of the fine river sand in the iron wok was monitored using an infrared thermometer (AR588, Smart Sensor, Dongguan, China). As the temperature was brought to 180 ± 5 °C, 200 g of raw glutinous rice grain was transferred into the iron wok and mixed with the sand and vigorously stirred at 180 ± 5 °C for 30 s. It was immediately removed from the hot sand by sieving (10 mesh) and spread on a marble slab for cooling [12]. The stir-fried glutinous rice after cooling was pulverized through using a grinder (ZX-2500Y, Yongkang Taihe Industry and Trade Co., Ltd., Yongkang, China) and then sieved through a 100-mesh sieve to obtain the cooked GRF, recorded as FRF.

For the heat–moisture treatment of GRF, the glutinous rice grain was subjected to heat–moisture treatment prior to grinding. The glutinous rice grain (200 g each) was put into a vacuum retort pouch and added with water by spraying the calculated amount of water evenly on the surface of the grain. The moisture contents of the samples were set to 12%, 18%, 24%, 30% and 36% (*w*/*w*). The pouch was sealed and left at a condition of 4 °C for 24 h. Then, the sample within the pouch was heated in an oven at 110 °C for 4 h. After cooling to room temperature, the HMT glutinous rice grain was taken out, and subsequently dried in an oven at 50 °C until the moisture content reached around 12% (*w*/*w*). Afterwards, the HMT glutinous rice grain was milled into flour with a grinder (ZX-2500Y, Yongkang Taihe Industry and Trade Co., Ltd., Yongkang, China) and sieved to 100 mesh to obtain HMT GRF, which were coded as HMT-12%, HMT-18%, HMT-24%, HMT-30% and HMT-36%, respectively. All samples were hermetically packaged in polyethylene bags and stored in a desiccator for further use.

#### 2.2.2. Preparation of Taopian

Taopian was prepared according to a traditional method [13]. Namely, ingredients including 100 g of maltose syrup, 100 g of icing sugar, 20 g of lard and 10 g of drinking water were mixed together with a blender for 10 min to generate a sticky batter. Then, 300 g of cooked GRF was blended with the batter for 15 min. After that, the mixture was transferred to a rectangular mold (136 mm × 78 mm × 38 mm). The loaded mixture was pressed with a rolling pin to form a compact cake. The compact cake was wrapped up with tinfoil and incubated in water bath at 55 ± 2 °C for 5 min. The compact cake was cut into pieces of approximately 2 mm thickness and left to cool to room temperature. Finally, the taopian samples were made and coded as FRF, HMT-12%, HMT-18%, HMT-24%, HMT-30% and HMT-36%, accordingly.

### 2.3. Characterization of the Cooked GRF and Taopian

#### 2.3.1. Color Measurements and Photo Acquisition

A spectrophotometer (CM-5, Konica Minolta, Osaka, Japan) was calibrated with a standard black-and-white tile and then was utilized to obtain the color values (L*, a* and b*) of the cooked GRFs and taopian samples. The whiteness index (*WI*) and the total color difference (Δ*E*) were calculated as the following Formulas (1) and (2), respectively:

(1) $$WI=100-\sqrt{{\left(a^{*}\right)}^{2}+{\left(b^{*}\right)}^{2}+{\left(100-L^{*}\right)}^{2}}$$

(2) $$∆E=\sqrt{{\left(∆L^{*}\right)}^{2}+{\left({∆a}^{*}\right)}^{2}+{\left({∆b}^{*}\right)}^{2}}$$

#### 2.3.2. Morphology and Particle Size of the Cooked GRF

The morphology of the cooked GRF samples was analyzed by using a scanning electron microscope (Phenom Pro, Phenom-World BV, Eindhoven, The Netherlands). Prior to the test, a small amount of sample was spread on double-sided conductive tape fixed to the sample stage, and sprayed with gold to increase the conductivity. The micrographs were acquired by operating at a voltage of 10 kV with 3000× magnification.

A laser diffraction particle size analyzer (Mastersizer 3000, Malvern, UK) was used to analyze the particle size of the rice flour samples. Prior to the assay, the specified amount of sample (1.0 g) was dispersed in deionized water and uniformly distributed by means of a vortex disperser, and then the feed solution was added to the sample cuvette until it reached 10% coverage. The particle size was expressed as D_(10)_, D_(50)_ and D_(90)_, the surface-weighted mean diameter (D[2,3]), the volume-weighted mean diameter (D[3,4]) and its distribution was expressed as Span = (D_(90)_ − D_(10)_)/D_(50)_.

#### 2.3.3. Fourier-Transform Infrared Spectroscopy (FTIR)

FTIR spectra of the cooked GRF samples were recorded using an FTIR spectrometer with an ATR accessory (Spectrum Two, Perkin-Elmer, Waltham, MA, USA). ATR-FTIR spectra between 4000 cm^−1^ and 400 cm^−1^ were recorded with a scan number of 4 and a resolution of 4 cm^−1^.

#### 2.3.4. Structural Characterization of Starch in the Cooked GRF

The X-ray diffraction patterns of the cooked GRF samples were explored by using an X-ray diffractometer (X’Pert3 Powder, Malvern Panalytical, Almelo, The Netherlands) with a Cu-Kα (λ = 0.154 nm) radiation source. The samples were placed on a square carrier, and measured at a target voltage of 40 kV and a current of 40 mA at a scanning rate of 2°/min. The diffractograms were recorded from 4° to 40° (2*θ*) with a scan step size of 0.013°. The relative crystallinity (RC) of the samples was analyzed using MDI Jade software (Version 6.0, Material Data Inc., Livermore, CA, USA) and calculated by the ratio of the area of crystalline peaks to the total diffraction area.

A small-angle X-ray scattering (SAXS) experiment was performed on a 1W2A beamline equipped with a two-dimensional Mar165 CCD detector at the Beijing Synchrotron Radiation Facility (BSRF) in China [14]. Prior to analysis, the sample was mixed with deionized water to make a slurry (50%, *w*/*w*), equilibrated at room temperature for 24 h and then placed in a sample holder with 2 mm thickness. The absolute intensity was calibrated with glass carbon and water [15]. The SAXS data were collected in the scattering vector (*q* = 4πsin*θ*/*λ*, 2*θ* was the scattering angle) range of 0.01–3 nm^−1^. FIT2D (v10.132) software was applied to convert the 2D scattering patterns to 1D scattering profiles. The data were collected and analyzed using the S.exe program written in Intel Visual Fortran [16].

#### 2.3.5. Moisture Content and Water Activity Measurements of the Cooked GRF

The moisture content (*MC*) of the GRF samples was determined according to the method of GB 5009.3-2016 [17]. Meanwhile, the water activity (*A*_w_) of the GRFs was determined according to the method of GB 5009.238-2016 [18] with an intelligent water activity tester (HD-3B, Wuxi Huake Instrument Co., Ltd., Wuxi, China).

#### 2.3.6. WAI, WSI and OBC Measurements of the Cooked GRF

The water absorption index (WAI) and water solubility index (WSI) of the cooked GRF were determined by the procedures described by Wang et al. [19] with some modifications. A mixture of GRF (2.0 g) and distilled water (40 mL) was added to a centrifuge tube and dispersed by a vortex stirring oscillator for 15 min at room temperature (25 °C). Followed by centrifuging at 4000 r/min for 15 min, the supernatant was dried to constant weight in a drying oven at 105 °C. The WAI and WSI of the flour samples were calculated by Equations (3) and (4).

(3) $$WAI=\frac{wet\;weight\;of\;sediment}{weight\;of\;the\;sample}\times 100\%$$

(4) $$WSI=\frac{dry\;weight\;of\;supernatant}{weight\;of\;the\;sample}\times 100\%$$

The oil-binding capacity (OBC) of the cooked GRF was determined by the method of Sun et al. [20]. Namely, 2.0 g of the sample was mixed with 20 mL of corn oil. After blending by a vortex stirring oscillator for 15 min, the mixture was centrifuged at 4000 r/min for 30 min at room temperature. Subsequently, the supernatant was decanted and the wet weight of the sediment was recorded. The OBC was computed by Equation (5).

(5) $$OBC=\frac{wet\;weight\;of\;sediment}{weight\;of\;the\;sample}\times 100\%$$

#### 2.3.7. Pasting Characterization of the Cooked GRF

The pasting properties of the cooked GRFs were determined by using a Rapid Visco Analyser (RVA-TecMaster, Perten Instruments, Warriewood, Australia) was used to determine. The cooked GRF (3.0 g) and deionized water (25.0 mL) were successively added into an aluminum RVA canister and stirred with a plastic paddle. The paddle rotating speed was held at 960 r/min during the first 10 s and then maintained at 160 r/min during the process, and the heating and cooling procedures of RVA were conducted in accordance with RVA Standard 1 [21]. The viscosity parameters were expressed in cP units.

#### 2.3.8. Low-Field Nuclear Magnetic Resonance of Taopian

The moisture distribution in taopian was analyzed by low-field nuclear magnetic resonance (LF-NMR) measurement using an LF-NMR system (MesoMR23-060H-I, Suzhou Niumag Analytical Instrument Co., Ltd., Suzhou, China). The taopian sample (5.00 g) was placed into an LF-NMR tube. The transverse relaxation time (*T*_2_) of taopian was determined using the Carr–Purcell–Meiboom–Gill (CPMG) sequence. The condition parameters were set as follows: the spectrometer frequency (SF) was 21 MHz, the time of echo (TE) was 0.1 ms, the echo count (NECH) was 18,000, the number of scans (NS) was 8 and the temperature was 32 °C.

#### 2.3.9. Textural Analysis of Taopian

The textural properties of taopian were determined using a texture analyzer (TA.XT Plus, Stable Micro Systems, Surrey, UK) with a P/36R probe in the texture profile analysis (TPA) mode. The samples of taopian were measured after cutting into a rectangular shape (70 × 30 mm^2^) with a thickness of 2 mm. The parameters were as follows: 1.0 mm/s test speed, 5.0 g trigger force and 30% compression ratio [22]. The pause between the first and the second compression test was 5 s. The parameters of the TPA including hardness, adhesiveness, cohesiveness and chewiness were recorded. Seven measurements were performed for each sample.

#### 2.3.10. Sensory Evaluation of Taopian

The sensory quality of taopian samples was evaluated by 20 panelists (10 males and 10 females, ages 20–25) from the College of Food Science, Southwest University. The protocol used in the present study was approved by the Ethical Committee of College of Food Science, Southwest University. Prior to the sensory evaluation, the panels were trained by using commercial taopian to get familiar with the terminology for each attribute of taopian and the use of a rating method [23]. The samples were randomly distributed along with a sensory evaluation questionnaire and presented to panelists. These samples were evaluated at room temperature for appearance, aroma, taste, hardness, stickiness, graininess and overall acceptability (Table A1 in Appendix A). Results were obtained using a 9-point hedonic scale (9 = like extremely, 8 = like very much, 7 = like moderately, 6 = like slightly, 5 = neither like nor dislike, 4 = dislike slightly, 3 = dislike moderately, 2 = dislike very much and 1= dislike extremely). The average score of each sensory attribute for the samples was calculated.

### 2.4. Statistical Analysis

All assays were performed at least in triplicate. The data were expressed as means ± standard deviation and analyzed using one-way analysis of variance (ANOVA) and means were compared by Duncan’s test with SPSS 26 software (IBMCorp., Armonk, NY, USA). Differences were considered to be significant at *p* < 0.05.

## 3. Results

### 3.1. Color, Morphology and Particle Size of Cooked GRF

**Figure 1:**
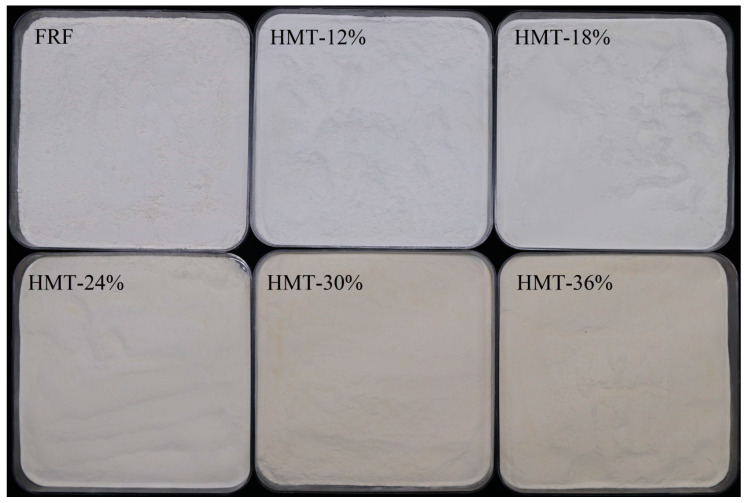
Digital photos of cooked glutinous rice flours. FRF: the flour made from stir-fried glutinous rice; HMT-12%, HMT-18%, HMT-24%, HMT-30% and HMT-36% represent the flours made from the glutinous rice subjected to heat–moisture treatment at the moisture content of 12%, 18%, 24%, 30% and 36%, respectively.

The photos of cooked GRFs are shown in Figure 1 and the color characteristics of FRF and HMT flours are shown in Table 1. HMT flours (except HMT-12%) showed significant lower brightness and were darker than FRF, as evidenced by significantly decreased L* and *WI* values. This was likely due to the chemical reaction in the low water content condition during the heat–moisture treatment. In addition, a* indicated the redness (+) and the greenness (–), and b* indicated the yellowness (+) and the blueness (–). FRF exhibited a slight greenness, while the HMT flours showed redness. All the samples showed yellowness. Moreover, a* and b* values increased with the increase in moisture levels for the HMT flours. The results indicated that the color of HMT flour became darker and browner as the moisture levels increased. This phenomenon was attributed to the probable promotion of the Maillard reaction during the heat–moisture treatment [24]. The color characteristics of HMT-30% and HMT-36% were markedly changed (Δ*E* = 8.98 and 10.85) compared with that of FRF. The color change between FRF and other HMT samples was also significantly different (*p* < 0.05). However, Δ*E* < 3 suggested that the color difference was not noticeable to the naked eye [25].

**Figure 2:**
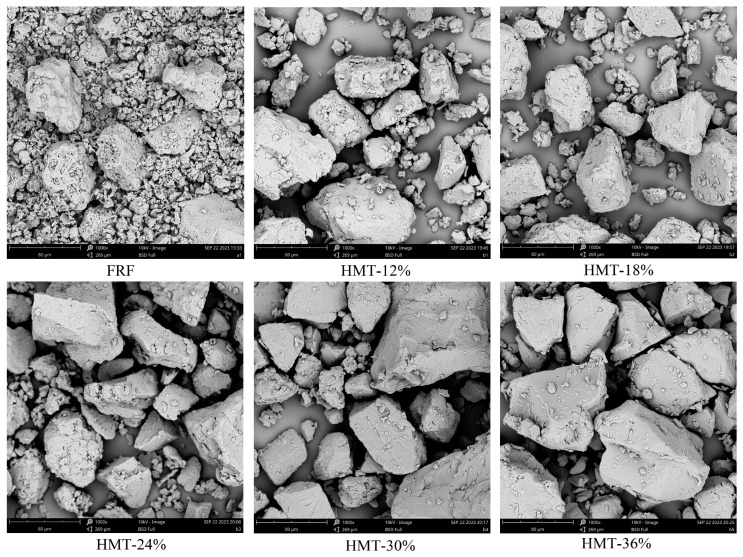
Scanning electron microscopy of cooked glutinous rice flours at 1000× magnification. FRF: the flour made from stir-fried glutinous rice; HMT-12%, HMT-18%, HMT-24%, HMT-30% and HMT-36% represent the flours made from the glutinous rice subjected to heat–moisture treatment at the moisture content of 12%, 18%, 24%, 30% and 36%, respectively. Scale bar = 80 μm.

The morphology of cooked GRFs is shown in Figure 2. For FRF, the surface of flour particles was densely covered with small holes, which was very different from the HMT flours. The main reason for this was that the moisture in the rice grain evaporated rapidly due to high temperature during the stir-frying process, escaping from the particles and forming pores. In addition, the typical angular or polyhedral structures of rice starch granules in FRF were largely invisible due the disintegration of the starch granules during stir-frying [26]. For the HMT flours, the morphology varied among the samples. With a low moisture level (12%, *w*/*w*), there were still relatively intact and agglomerate rice starch granules in the particles. However, the structural integrity of the rice starch granules was almost lost as the moisture levels increased to higher values (18–36%, *w*/*w*). This indicated that partial gelatinization of the starch granules in the HMT flours and the elevated moisture level accelerated this process [26]. Due to the greater extent of starch gelatinization at higher moisture levels, the HMT-30% and HMT-36% had a more even surface and more compact particles compared with other samples.

**Figure 3:**
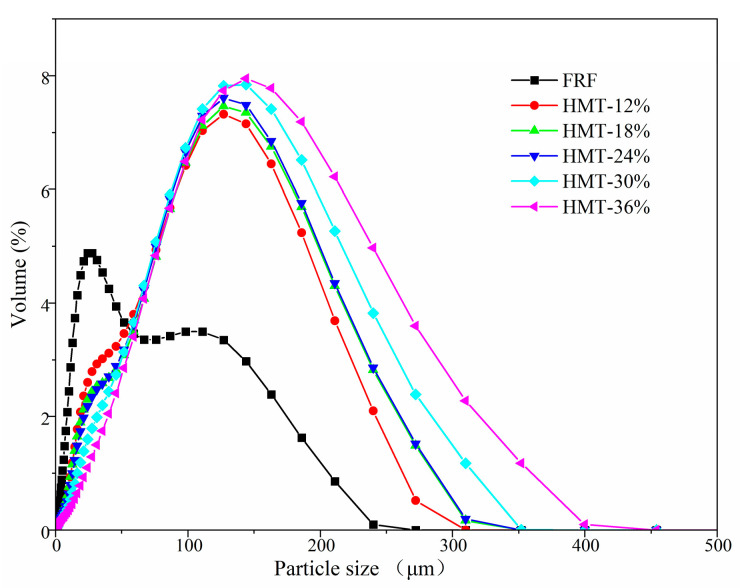
Particle size distribution of cooked glutinous rice flours. FRF: the flour made from stir-fried glutinous rice; HMT-12%, HMT-18%, HMT-24%, HMT-30% and HMT-36% represent the flours made from the glutinous rice subjected to heat–moisture treatment at the moisture content of 12%, 18%, 24%, 30% and 36%, respectively.

From the particle size distribution plotted in Figure 3, between FRF and HMT flours a notable distinction in particle size distribution was observed. The particle size distribution curve of FRF was bimodal, with a shoulder peak at around 110 μm. However, the curves of HMT flours were almost unimodal [27]. As listed in Table 1, D_(50)_, D[3,4] and D[2,3] values of HMT flours were greater than those of FRF. This indicated that HMT rice grains were more resistant to grinding into the flours. In addition, Span = (D_(90)_ − D_(10)_)/D_(50)_, an indication of the volume-based size distribution width of the particle, showed that HMT flours exhibited a narrower particle size distribution than FRF [28].

### 3.2. Fourier-Transform Infrared Spectroscopy (FTIR)

**Figure 4:**
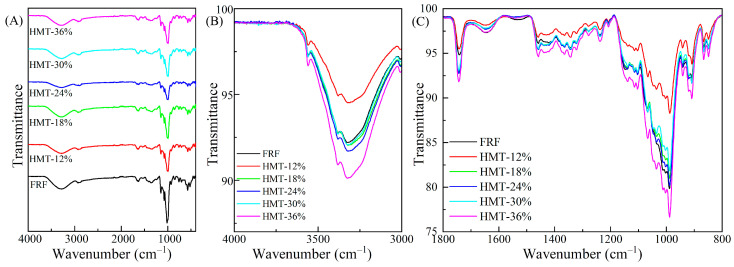
FT-IR spectra of the cooked glutinous rice flours: (**A**) FTIR spectra at 4000–400 cm^−1^; (**B**) FT-IR spectra at 4000–3000 cm^−1^; and (**C**) FT-IR spectra at 1800–800 cm^−1^. FRF: the flour made from stir-fried glutinous rice; HMT-12%, HMT-18%, HMT-24%, HMT-30% and HMT-36% represent the flours made from the glutinous rice subjected to heat–moisture treatment at the moisture content of 12%, 18%, 24%, 30% and 36%, respectively.

FTIR analysis was applied to investigate the vibrational modes of functional groups of the rice flour components. The FTIR spectra of the cooked glutinous rice flours are depicted in Figure 4A–C. As indicated in Figure 4C, all the samples exhibited almost similar signature but different band intensities, indicating that to large extent both FRF and HMT flours showed similar chemical compositions [29]. The skeleton of starch molecules is mainly composed of –OH, –C-OH, –CH_2_- and –CH-groups. The broad bands at 3000–3600 cm^−1^ were related to inter- and intramolecular hydrogen bonding and hydroxyl (O-H) stretching vibrations [30]. As examined closely, the remarkable difference observed in this region (Figure 4B), other than the band shift, was the variation in intensity (expressed as transmittance). Except for HMT-12%, other HMT flours showed an equal or stronger intensity of hydrogen stretching bands compared with FRF. This indicated that heat–moisture treatment led to an enhancement of hydrogen bonding within the cooked GRFs. The bands at 2920 cm^−1^ and 2847 cm^−1^ were associated with the C-H stretching vibrations. The bands at 1630–1660 cm^−1^, 1100–1150 cm^−1^, and 1100–900 cm^−1^ were assigned to carbonyl (–C-OH) stretching, C–C stretching, and –C-O–H bending, respectively. In addition, the bands at 1742 cm^−1^ and 1532 cm^−1^ represented the C=O stretching vibration of the protein peptide group and protein amide II band [31]. As shown in Figure 4C, the protein absorption band at 1742 cm^−1^ of the HMT samples that were treated at high moisture levels was strengthened compared with FRF. In addition, the bands in the region of 1100–900 cm^−1^ were associated with the interaction between starch molecules and non-starch components, such as proteins. The marked variation in intensity of this region was probably due to the effect of heat–moisture treatment on the interaction of starch molecules with non-starch components in the glutinous rice flour.

### 3.3. Structural Characteristics of Starch in the Cooked GRF

#### 3.3.1. Crystalline Structure of Starch in the Cooked GRF

**Figure 5:**
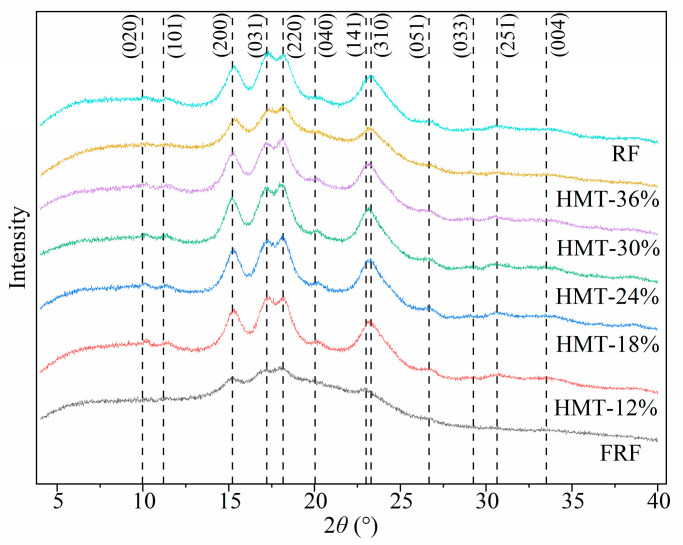
X-ray diffraction patterns of cooked glutinous rice flours. RF: raw glutinous rice flour. FRF: the flour made from stir-fried glutinous rice; HMT-12%, HMT-18%, HMT-24%, HMT-30% and HMT-36% represent the flours made from the glutinous rice subjected to heat–moisture treatment at the moisture content of 12%, 18%, 24%, 30% and 36%, respectively.

The X-ray diffraction (XRD) patterns of raw rice flour and cooked rice flour (FRF and HMT) samples are shown in Figure 5. The rice flour showed diffraction peaks at 2*θ* of 15°, 17°, 18° and 23°, indicating A-type starch in the rice flour [32]. According to a previous study from Rodriguez-Garcia et al. [33], the dotted lines correspond to the identification of the orthorhombic crystal structure in glutinous rice starch (Figure 5). Meanwhile, the Miller indexes (*hkl*), Bragg angles (2*θ*) and interplanar spacings (*d*) for the orthorhombic phase in the rice starch of the glutinous flour samples are listed in Table A2. By comparing the XRD patterns of GRFs and other cereal samples, it was confirmed that the starch in GRFs presented an orthorhombic crystal structure [34]. The cooking process, i.e., both stir-frying and heat–moisture treatment, did not alter the crystal structure of the starch in the cooked rice flours. However, the relative crystallinity (RC) of the starch in each rice flour sample varied, as listed in Table 2. The RC of FRF was 19.92%, which was markedly decreased when compared with that of raw rice flour (28.07%). The HMT flours (except HMT-36%) showed greater RC values than the raw rice flour. These results implied that the starch structure became more organized in HMT rice flours. Due to the plasticization effect of water molecules at the HMT temperature (110 °C), it was probably that HMT facilitated the formation of double helix structures of amylose and amylopectin chains in the amorphous region. Consequently, a greater RC was found in these samples when undergoing HTM processes at the low moisture levels (12%, 18% and 24%). A reduction in the RC of the cooked flour HMT-36% was observed, however, in which the gelatinization of the starch was more dominant at higher moisture levels (36%, *w*/*w*) [35]. Lv et al. [36] reported that HMT-treated highland barley flour at moisture levels 15%, 25% and 35% retained the orthorhombic crystal structure of the starch, whereas the RC of the starch in the flour increased from 28.52% to 41.32%.

#### 3.3.2. Lamellar Structures of Starch in the Cooked GRF

**Figure 6:**
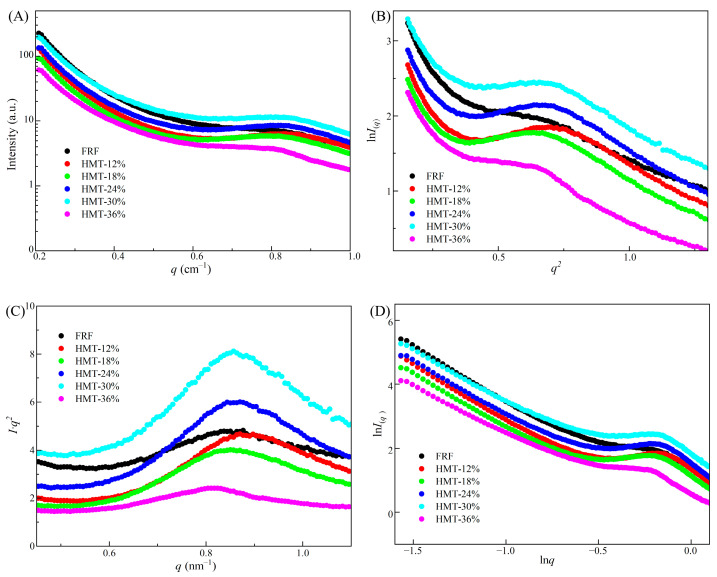
(**A**) Peak curves of SAXS patterns of cooked glutinous rice flours; (**B**) Guinier SAXS plots of cooked glutinous rice flours; (**C**) Lorentz transformation of cooked glutinous rice flours; (**D**) double-logarithmic SAXS plots of cooked glutinous rice fours. FRF: the flour made from stir-fried glutinous rice; HMT-12%, HMT-18%, HMT-24%, HMT-30% and HMT-36% represent the flours made from the glutinous rice subjected to heat–moisture treatment at the moisture content of 12%, 18%, 24%, 30% and 36%, respectively.

The lamellar structure difference in starch in the rice flours with stir-frying or heat–moisture treatment was examined by using SAXS. Figure 6A depicts the SAXS pattern of the scattering intensity *I* versus scattering vector *q*. It was shown that a typical scattering peak around 0.82~0.84 nm^−1^ was found in all samples. The visible scattering peak revealed the lamellar repeat distance in starch granules [37]. Further, to calculate the radius of gyration (*R_g_*) of the lamellar structure of glutinous rice starch, the SAXS profile in Figure 6A was fitted by Guinier approximation and Porod analysis ($I_{\left(q\right)}={\;I}_{\left(0\right)}\;exp(-R_{g}^{2}\;q^{2}/3)$). A Guinier plot (ln(*I*_(*q*)_)~*q*^2^) is depicted in Figure 6B, and the radius of gyration ($R_{g}=\sqrt{-3\beta }$) was obtained by using the initial slope (*β*) of the plots [38]. The *R_g_* value of FRF was 2.11 nm, while the *R_g_* value of HMT flours varied in the range of 1.86~2.14. The periodic thickness (*d*_Bragg_) of the semi-crystalline lamellae could be calculated by Woolf Bragg’s equation (*d*_Bragg_ = $2\pi /q^{*}$), while q* (nm^−1^) was the peak position in the Lorentz-corrected SAXS profiles (Figure 6C). The *d*_Bragg_ value of FRF was 7.17 nm. HMT flours had a greater *d*_Bragg_ value than FRF (Table 2). Moreover, fractals are a class of self-similar structures with no feature length, and the quantitative characterization parameter of their irregularity is the fractal dimension (D). The scattering patterns from a fractal object generally obey a power law equation in the low-*q* region, *I* ∝ *q*^α^, where the fractal structure is revealed by the exponent *α*, which can be calculated from the slope of double-logarithmic SAXS patterns ln(*I*(*q*))~ln*q* (Figure 6D). The scattering object is the surface fractal dimension (D*_s_* = 6+α) if −4<α<−3, whereas the fractal dimension is a mass fractal structure with a D*_m_* =−α if −3<α<−1. As shown in Table 2, the starch in HMT rice flours showed a mass fractal dimension (D*_m_*) in the range of 1.77–2.04, which was lower than that in FRF (D*_m_* =2.28). The results indicated that the starch in FRF had greater compactness and a denser inner structure compared to that in HMT rice flours [39]. By the correlation function analysis performed using the S.exe (v05.05) program package [16], the long-period distance (*L*, nm), the average thickness of the amorphous lamellae (*d*_a_, nm) and the average thickness of the crystalline lamellae (*d*_c_ = *L* − *d*_a_, nm) was calculated. The *L* and *d_a_* values of HMT flours were greater than those of FRF.

### 3.4. Physicochemical Properties of the Cooked GRF

#### 3.4.1. Water Content, Water Activity and Hydration Properties

As shown in Table 3, FRF had the lowest moisture content among the samples. The moisture content of HMT rice flours was varied, depending on the moisture level during the heat–moisture treatment. As the HMT moisture level decreased, more moisture (from 11.49% to 12.79%) was retained in the flour. The results indicated that the interaction between the water and the flour components was strengthened by HMT. The water activity (*A_W_*) of FRF was 0.631, whereas the *A_W_* of HMT rice flours was in the range of 0.589–0.627. This phenomenon was probably due to the enhanced interaction between the water and the flour components in HMT flours. It was further supported by WAI and WSI, as HMT flours had markedly lower WAI and WSI values than FRF (Table 3). Structurally, FRF showed small holes on the surface of the particles, having a smaller mean size (D_(50)_) and a markedly lower degree of crystallinity. These characteristics were more helpful for water soaking into the flour particles. Conversely, the HMT flours had a greater degree of crystallinity, larger mean size and compact nature of the particles, which hindered the water absorption and migration [40]. In addition, HMT restructured the starch molecules into more ordered clusters, which elevated the interaction among the starch molecules. Therefore, HMT rice flours had lower WSI values than FRF. Similarly, Kumar et al. [41] reported that HMT (110 °C for 4 h; 25% moisture content) decreased the swelling power and solubility of proso millet flour and starch.

#### 3.4.2. Oil Binding

The oil-binding capacity results are shown in Table 3. The results showed that the GRF oil-binding capacity ranged from 88.6% to 105.8%. Namely, the oil-holding capacity of FRF, HMT-12%, HMT-18% and HMT-24% showed no significant difference, while the oil-holding capacity significantly decreased when the HMT moisture level was 30% and 36%. Zhou et al. [42] found that dry-heating-treated rice flour (120 °C for 120 min in an oven) had a higher oil-binding capacity than the control. The authors concluded that the increased oil-binding capacity was probably due to the enhancement of hydrophobicity of rice protein through dry-heating treatment. Similar results were found in the study reported by Zhu et al. [43], who found that the increase in the oil-binding capacity of rice starch was more pronounced under the combination of dry heating with whey protein isolate. In our study, however, the oil-binding capacity of the cooked flours was largely related to the microstructure and chemical components of the particles. HMT-30% and HMT-36% had larger particle sizes, smoother surfaces and denser structures of the particles than other samples, which were not favorable for oil binding.

#### 3.4.3. Pasting Properties

**Figure 7:**
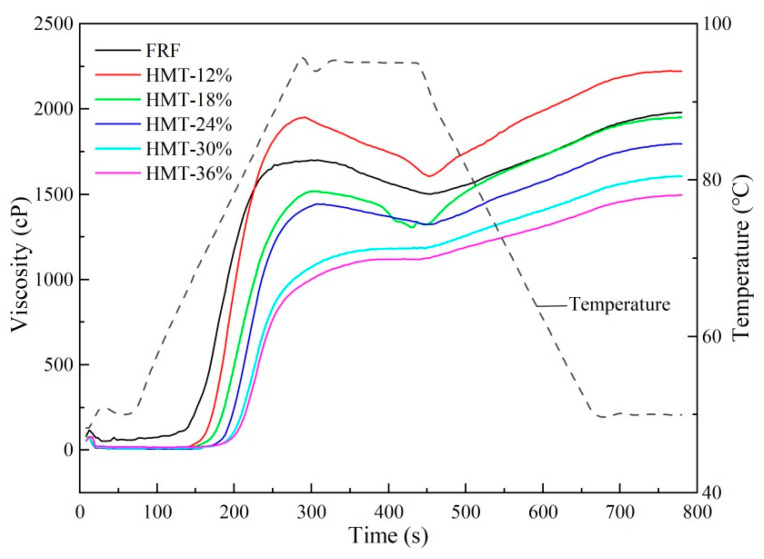
Rapid viscosity profiles of cooked glutinous rice flours. FRF: the flour made from stir-fried glutinous rice; HMT-12%, HMT-18%, HMT-24%, HMT-30% and HMT-36% represent the flours made from the glutinous rice subjected to heat–moisture treatment at the moisture content of 12%, 18%, 24%, 30% and 36%, respectively.

The pasting profiles of FRF and HMT rice flour samples are shown in Figure 7, while the corresponding pasting parameters are given in Table 3. The pasting temperature (PT) is the temperature at which the viscosity of mixture started to rise. It showed that HMT flours had a greater PT than FRF, indicating a higher resistance to swelling of the starch granules [44]. In addition, the pasting profiles showed that the peak viscosity (PV), trough viscosity (TV) and final viscosity (FV) of HMT-12% were significantly greater than those of RFR, whereas the PV, TV and FV values of other HMT-treated samples were lower than those of FRF. These differences are probably a result of the re-organization of starch molecules during stir-frying or heat–moisture treatment. After heat–moisture treatment at moisture levels 18%~36% and recrystallization upon cooling and drying, the starch in HMT-treated samples was expected to form a more compact structure. The starch granules were less swelled upon heating before physical rupture, and therefore, a decrease in PV was observed in HMT-treated samples (except HMT-12%). FV measured the ability of the gelatinized starch to form a viscous paste after heating and cooling to 50 °C. It was indicated that HMT could reduce the amylose leaching during the RVA test. The TV is the minimum viscosity between the PV and FV. The difference between the PV and TV is called the breakdown (BD) viscosity, which reflects the stability of hot starch paste [41]. The decrease in the BD as the moisture level increased from 12% to 36% indicated that HMT strengthened the internal interactions between the components in the rice flours [45]. The setback (SB) viscosity indicates the tendency of the starch paste to retrograde. As the moisture level increased, the SB values for HMT-treated samples gradually decreased.

### 3.5. Physicochemical Properties of Taopian

#### 3.5.1. Appearance and Color of Taopian

**Figure 8:**
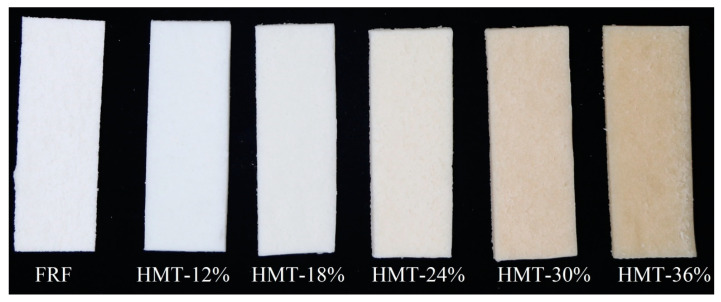
Digital photos of taopian samples. FRF represents taopian made with the flour made from stir-fried glutinous rice; HMT-12%, HMT-18%, HMT-24%, HMT-30% and HMT-36% represent taopian made with the flours made from the glutinous rice subjected to heat–moisture treatment at the moisture content of 12%, 18%, 24%, 30% and 36%, respectively.

As shown in Figure 8, the taopian made of GRFs with different pre-treatments (stir-frying and heat–moisture treatment) showed a uniform and clean external appearance, with a smooth and glossy surface. The taopian made of FRF was off-white, reflecting the color of the flour used. The taopian samples made of HMT-treated flours were off-white or creamy-white to beige. The color parameters of taopian are presented in Table 4. The taopian samples made of HMT-12% showed similar L* and *WI* values to those made of FRF. As the moisture levels increased during HMT, the L* and *WI* values of taopian significantly decreased. The results were persistent with the change in L* and *WI* values of the cooked GRFs, due to the flours being the main ingredient for preparing taopian. In addition, an increase in a* and b* was observed in the taopian made of HMT flours with increasing moisture level during the heat–moisture treatment. This was mainly due to the Maillard reaction during the pre-treatment of the glutinous rice materials. In previous studies, it has been demonstrated that the color differences indicated by Δ*E* > 5 can be discriminated obviously [46]. Hence, the color of taopian made of HMT-24%, HMT-30% and HMT-36% was significantly different from that of taopian made of FRF.

#### 3.5.2. Water Distribution of Taopian

**Figure 9:**
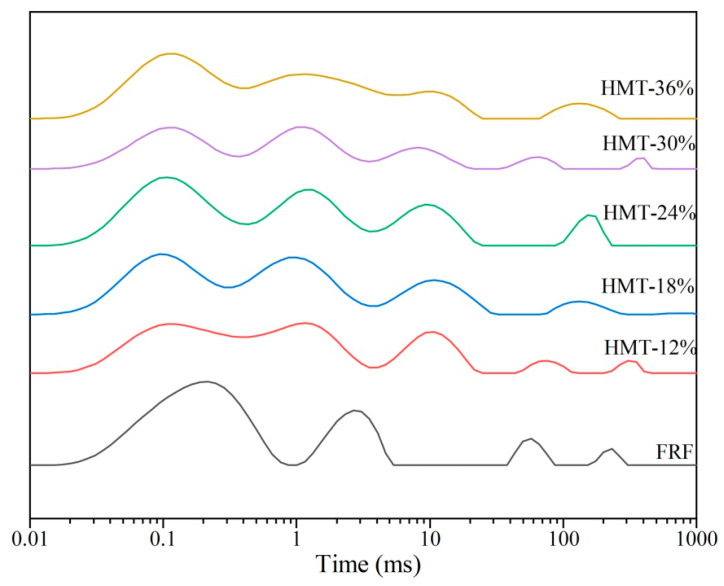
*T*_2_ inversion map of taopian samples. FRF represents taopian made with the flour made from stir-fried glutinous rice; HMT-12%, HMT-18%, HMT-24%, HMT-30% and HMT-36% represent taopian made with the flours made from the glutinous rice subjected to heat–moisture treatment at the moisture content of 12%, 18%, 24%, 30% and 36%, respectively.

Figure 9 shows the hydrogen proton transverse relaxation time (*T*_2_) distribution curves of the taopian samples. The contents of water and oil in the taopian samples were 14.28% and 3.77%, respectively. Therefore, the proton fractions both contributed to water and oil. According to references [47,48], the proton fraction of *T*_21_ (0.01~1 ms) corresponded to bound water, *T*_22_ (1~40 ms) represented the relaxation time of immobile water and fat (the lard), and *T*_23_ (40~500 ms) referred to free water. The order of the area of each proton fraction in descending order was *T*_21_ > *T*_22_ > *T*_23_, indicating that the proportion of the free water was quite small in the taopian samples. The peak of *T*_21_ was 0.22 ms for the taopian made with FRF, whereas the *T*_21_ peak for the taopian made with HMT flour shifted to a shorter relaxation time (0.09~0.12 ms). The shorter *T*_21_ indicated that the water bound more tightly with the components in HMT flours. The peak (*T*_22a_) that was next to *T*_21_ was described as immobile water, and the peak (*T*_22b_) centered around 8~10 ms corresponded to the lard [49]. For the taopian made with FRF, the peak *T*_22a_ was merged with *T*_21_, implying that the immobile water in FRF sample was located in a more fixed micro-environment than in HMT flours [50]. In addition, *T*_23_ had the longest relaxation time and this part of water had molecular fluidity in an aqueous solution. However, the proportion for this part was quite small, accounting for around 4.5%~8.0% of the total peak area.

#### 3.5.3. Textural Properties of Taopian

The texture characteristics (hardness, adhesiveness, cohesiveness and chewiness) of taopian are shown in the Table 4. Hardness was the maximal force needed to distort the taopian sample during the first compression. A moderate hardness is favorable for the taopian’s palatability. The taopian made of HMT-12% had the lowest hardness. The hardness of HMT flours increased as the moisture level during heat–moisture treatment increased. The results indicated that the structural integrity of taopian could be favored by using HMT flours at increased moisture levels. The adhesiveness indicated the ability of the sample to adhere to the surface of the objects (the probe or teeth), reflecting the surface properties of the sample [51]. Adhesiveness was calculated using the area under the negative stress–strain curve following the first compression. The area of the peak indicated the measure of work needed to overcome the adhesive forces between the sample and the probe during the withdrawal of the probe. Compared with taopian made of FRF, the adhesiveness values of taopian made with HMT rice flours increased with the increment of moisture levels. The adhesiveness of sample HMT-12% was 29.40 and for HMT-36% it was 86.50, indicating that HMT-36% was more adhesive than HMT-12% because the area of the negative peak was higher in the case of HMT-36%. This implied that appropriate HMT flour could enhance the palatability of taopian with a moderate adhesiveness. In addition, cohesiveness reflected the extent to which the taopian’s structure was disrupted during first compression. The cohesiveness of taopian made with HMT rice flours increased as the moisture level increased during HMT treatment. Chewiness was a measure of the force required to cause a specific degree of deformation in the sample. The chewiness of taopian made of HMT-12% was lower, whereas that of HMT-30% and HMT-36% was higher than that of taopian made of FRF.

### 3.6. Sensory Properties of Taopian

**Figure 10:**
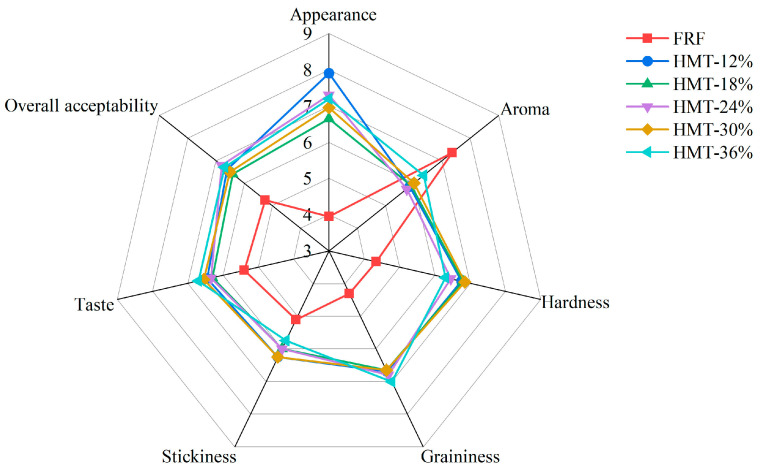
Sensory evaluation of taopian samples. FRF represents taopian made with the flour made from stir-fried glutinous rice; HMT-12%, HMT-18%, HMT-24%, HMT-30% and HMT-36% represent taopian made with the flours made from the glutinous rice subjected to heat–moisture treatment at the moisture content of 12%, 18%, 24%, 30% and 36%, respectively.

Taopian commonly presents good appearance, flavor and palatability. Structurally, taopian is a sliced pastry made of cooked GRF with syrup and lard as the binders. Therefore, its good appearance includes a high integrity of the slice with a smooth and glossy surface; its good flavor covers the unique aroma of cooked glutinous rice and taste of the ingredients; and its good palatability is composed of moderate hardness (softness), graininess and stickiness. As shown in Figure 10, the taopian samples made of HMT flours received notably higher scores across nearly all sensory attributes (except aroma) compared to the FRF group. The taopian made of HMT-12% obtained the highest appearance score (7.9), while FRF had the lowest (4.0). The aroma score of FRF surpassed that of the HMT flours, indicating pronounced aroma development in glutinous rice during the stir-frying procedure which was likely associated with the Maillard reaction. Nonetheless, taopian samples made of HMT flours had elevated scores in flavor from a holistic perspective, including the whole taste and aroma. According to the panel, the taopian samples made of HMT-36% were the most preferred, with a moderate hardness and not sticky feeling in the oral cavity. In addition, the overall acceptability of FRF was 5.25. However, HMT flours ranged from 6.4 to 6.8 (a maximum of 9 points), with HMT-18% receiving the lowest score (6.4) and HMT-24% garnering the highest overall acceptance (6.8). In summary, the use of heat–moisture-treated GRF instead of stir-frying GRF could improve the sensory quality of taopian to a certain extent.

## 4. Conclusions

The present study compared the physicochemical properties associated with stir-fried or heat–moisture-treated GRFs and the quality of taopian made thereof. The GRFs exhibited water activity in the range of 0.589–0.631. The HMT flours had lower whiteness indexes, greater mean particle sizes, and lower water absorption indexes and water solubility indexes than FRF. Furthermore, heat–moisture treatment had a far greater impact on the relative crystallinity and lamellar structure of the starch in GRFs than stir-frying. As the moisture content during the heat–moisture treatment increased, the breakdown and setback of the flours decreased, indicating a good stability of HMT flours. Accordingly, the taopian made with FRF or HMT flours exhibited differences in color, texture, water distribution and sensory scores. Low-field nuclear magnetic resonance indicated that bound and immobile water dominated in the taopian samples. More tightly bound water was observed in the taopian made with HMT flours than FRF. Moreover, the taopian samples prepared from GRF with appropriate heat–moisture treatment showed better textural and sensory qualities, such as moderate hardness, adhesiveness and higher overall acceptance. These results implied that heat–moisture treatment is a promising way to improve the physicochemical properties of cooked GRFs and the quality of taopian made with the corresponding flours.

## Figures and Tables

**Table 1:** Color and particle size of rice flours by different cooking methods.

	FRF	HMT-12%	HMT-18%	HMT-24%	HMT-30%	HMT-36%
Color						
L*	96.34 ± 0.14 a	96.26 ± 0.13 a	95.52 ± 0.31 b	95.25 ± 0.08 c	91.91 ± 0.11 d	90.99 ± 0.08 e
a*	−0.05 ± 0.01 f	0.33 ± 0.02 e	0.62 ± 0.02 c	0.42 ± 0.01 d	2.16 ± 0.07 b	2.62 ± 0.04 a
b*	5.37 ± 0.03 e	7.76 ± 0.19 d	8.55 ± 0.08 c	8.35 ± 0.26 c	12.86 ± 0.23 b	14.43 ± 0.14 a
*WI*	93.50 ± 0.10 a	91.38 ± 0.22 b	90.32 ± 0.08 c	90.38 ± 0.26 c	84.65 ± 0.25 d	82.79 ± 0.14 e
Δ*E*	—	2.43 ± 0.21 d	3.37 ± 0.08 c	3.21 ± 0.19 c	8.98 ± 0.34 b	10.85 ± 0.13 a
Particle size						
D_(10)_	9.02	18.9	18.8	20.8	26.7	34.7
D_(50)_	33.7	89.5	97.3	99.3	111	124
D_(90)_	133	193	205	206	225	253
Span	3.68	1.95	1.91	1.87	1.79	1.76
D[3,4]	53.7	97.8	105	107	119	136
D[2,3]	20.2	37.2	37.9	40.4	49.6	60.7

Note: *WI*, whiteness index; Δ*E*, the overall color difference. FRF: the flour made from stir-fried glutinous rice; HMT-12%, HMT-18%, HMT-24%, HMT-30% and HMT-36% represent the flours made from the glutinous rice subjected to heat–moisture treatment at the moisture content of 12%, 18%, 24%, 30% and 36%, respectively. The values are expressed as the mean ± standard deviation of triplicate experiments. Values in the same line with different letters indicate significant difference (*p* < 0.05).

**Table 2:** Structural characteristics of rice flours obtained by different cooking methods.

	FRF	HMT-12%	HMT-18%	HMT-24%	HMT-30%	HMT-36%
RC (%)	19.92 ± 2.30 d	42.61 ± 2.94 a	42.92 ± 3.13 a	41.22 ± 2.05 a	36.67 ± 2.92 b	23.08 ± 2.79 d
*R*_g_ (nm)	2.11 ± 0.02 b	1.86 ± 0.00 e	1.92 ± 0.02 d	1.93 ± 0.01 d	2.00 ± 0.01 c	2.14 ± 0.00 a
D*_m_*	2.28 ± 0.00 a	1.86 ± 0.01 c	1.80 ± 0.01 d	1.77 ± 0.04 d	1.84 ± 0.01 c	2.04 ± 0.00 b
*d*_Bragg_ (nm)	7.17 ± 0.00 c	7.22 ± 0.00 c	7.35 ± 0.03 b	7.33 ± 0.11 b	7.33 ± 0.06 b	7.75 ± 0.06 a
*d*_c_ (nm)	2.54 ± 0.01 b	2.45 ± 0.00 e	2.49 ± 0.01 c	2.48 ± 0.00 d	2.49 ± 0.00 cd	2.63 ± 0.00 a
*d*_a_ (nm)	3.01 ± 0.00 f	3.23 ± 0.00 e	3.36 ± 0.00 b	3.29 ± 0.00 d	3.30 ± 0.00 c	3.43 ± 0.00 a
*L* (nm)	5.55 ± 0.01 f	5.68 ± 0.00 e	5.85 ± 0.01 b	5.77 ± 0.00 d	5.79 ± 0.01 c	6.06 ± 0.01 a

Note: RC, relative crystallinity; *R_g_*, rotation radius; D*_m_*, the mass fractal dimension; *d*_Bragg_, Bragg spacing or lamellar repeat distance; *d*_c_, the thickness of the crystalline lamellar; *d*_a_, the thickness of the amorphous lamellar; *L*, the long period distance. FRF: the flour made from stir-fried glutinous rice; HMT-12%, HMT-18%, HMT-24%, HMT-30% and HMT-36% represent the flours made from the glutinous rice subjected to heat–moisture treatment at the moisture content of 12%, 18%, 24%, 30% and 36%, respectively. The values are expressed as the mean ± standard deviation of triplicate experiments. Values in the same line with different letters indicate significant difference (*p* < 0.05).

**Table 3:** Physicochemical properties of rice flours obtained by different cooking methods.

	FRF	HMT-12%	HMT-18%	HMT-24%	HMT-30%	HMT-36%
Physical properties						
*MC*	10.97 ± 0.02 f	12.79 ± 0.01 a	12.35 ± 0.02 b	12.25 ± 0.01 c	12.00 ± 0.02 d	11.49 ± 0.04 e
*A_w_*	0.631 ± 0.013 a	0.627 ± 0.018 a	0.602 ± 0.006 bc	0.623 ± 0.017 ab	0.617 ± 0.002 ab	0.589 ± 0.004 c
WAI (%)	301.7 ± 28.2 a	150.2 ± 1.7 c	148.5 ± 1.0 c	144.6 ± 2.1 c	163.1 ± 1.0 c	185.9 ± 2.2 b
WSI (%)	6.1 ± 0.6 a	3.6 ± 0.2 b	3.5 ± 0.1 b	3.5 ± 0.2 b	3.5 ± 0.1 b	1.2 ± 0.5 c
OBC (%)	105.8 ± 2.7 a	102.2 ± 1.8 ab	100.2 ± 4.7 ab	100.4 ± 5.9 ab	96.9 ± 1.9 b	88.6 ± 1.1 c
Pasting properties						
PT (°C)	60.67 ± 8.77 c	69.35 ± 0.05 b	72.03 ± 0.41 ab	74.78 ± 0.51 ab	76.95 ± 0.48 a	77.68 ± 0.45 a
PV (cP)	1706.67 ± 34.36 b	1953.33 ± 10.60 a	1519.67 ± 21.96 c	1444.33 ± 12.34 d	1186.00 ± 35.76 e	1126.33 ± 18.82 f
TV (cP)	1502.33 ± 27.43 b	1607.00 ± 21.93 a	1297.33 ± 64.04 c	1322.67 ± 21.01 c	1168.33 ± 28.57 d	1101 ± 3.61 e
FV (cP)	1980.67 ± 10.21 b	2222.33 ± 10.12 a	1952.00 ± 12.12 b	1797.00 ± 8.72 c	1606.33 ± 45.24 d	1495.33 ± 7.09 e
BD (cP)	204.33 ± 31.56 b	346.33 ± 16.62 a	222.33 ± 65.99 b	121.67 ± 9.29 c	17.67 ± 7.37 d	25.33 ± 16.20 d
SB (cP)	478.33 ± 22.37 b	615.33 ± 29.09 a	654.67 ± 71.86 a	474.33 ± 23.86 b	438.00 ± 16.70 bc	394.33 ± 10.69 c

Note: *MC*: moisture content; *A_w_*: water activity; WAI: water absorption index; WSI: water solubility index; OBC: oil-binding capacity; PT: pasting temperature; PV: peak viscosity; TV: trough viscosity; FV: final viscosity; BD: breakdown value; SB: setback value. FRF: the flour made from stir-fried glutinous rice; HMT-12%, HMT-18%, HMT-24%, HMT-30% and HMT-36% represent the flours made from the glutinous rice subjected to heat–moisture treatment at the moisture content of 12%, 18%, 24%, 30% and 36%, respectively. The values are expressed as the mean ± standard deviation of triplicate experiments. Values in the same line with different letters indicate significant difference (*p* < 0.05).

**Table 4:** Color and textural characteristics of taopian made from rice flours obtained by different cooking methods.

	FRF	HMT-12%	HMT-18%	HMT-24%	HMT-30%	HMT-36%
Color						
L*	88.15 ± 0.56 a	87.74 ± 0.41 a	86.68 ± 0.37 b	84.08 ± 0.34 c	77.84 ± 0.60 d	71.89 ± 0.93 e
a*	0.13 ± 0.16 c	−1.13 ± 0.15 e	−0.44 ± 0.16 d	1.96 ± 0.60 b	4.35 ± 0.49 a	4.58 ± 0.48 a
b*	10.68 ± 0.53 e	9.67 ± 0.67 f	12.81 ± 0.97 d	17.44 ± 0.82 c	21.97 ± 0.51 b	24.42 ± 0.23 a
WI	84.04 ± 0.70 a	84.33 ± 0.23 a	81.50 ± 0.76 b	76.29 ± 0.58 c	68.49 ± 0.66 d	62.48 ± 0.67 e
Δ*E*	—	1.89 ± 0.50 d	2.81 ± 1.15 d	8.14 ± 1.23 c	15.87 ± 1.05 b	21.76 ± 1.08 a
Textural characteristics						
Hardness (g)	13,139 ± 2557 c	10,343 ± 1999 d	15,955 ± 3071 ab	14,054 ± 2364 bc	18,133 ± 2893 a	17,398 ± 1998 a
Adhesiveness (g·s)	24.77 ± 18.32 d	29.40 ± 9.89 d	46.71 ± 12.11 bc	37.38 ± 9.78 cd	57.14 ± 14.78 b	86.50 ± 14.37 a
Cohesiveness	0.74 ± 0.05 a	0.57 ± 0.06 c	0.67 ± 0.06 b	0.67 ± 0.05 b	0.73 ± 0.05 a	0.71 ± 0.03 ab
Chewiness	6607.8 ± 2224.6 b	3613.2 ± 1216.0 c	6832.1 ± 2138.6 ab	5615.2 ± 1581.1 b	8444.5 ± 1716.9 a	8361.6 ± 1466.6 a

Note: *WI*, whiteness index; Δ*E*, the overall color difference; FRF represents taopian made with the flour made from stir-fried glutinous rice; HMT-12%, HMT-18%, HMT-24%, HMT-30% and HMT-36% represent taopian made with the flours made from the glutinous rice subjected to heat–moisture treatment at the moisture content of 12%, 18%, 24%, 30% and 36%, respectively. The values are expressed as the mean ± standard deviation of triplicate experiments. Values in the same line with different letters indicate significant difference (*p* < 0.05).

## Data Availability

The original contributions presented in the study are included in the article, further inquiries can be directed to the corresponding author.

## Appendix A

**Table A1:** Sensory scoring items for taopian samples.

Evaluation Index	Specific Feature Description
Appearance	Normal color, dense and uniform surface structure (7–9)
	White or slightly yellow, slightly loose structure, no crack (4–6)
	Brown or ustulate, loose and rough structure with cracks (1–3)
Aroma	Pleasant scent, a distinct unique smell of cooked glutinous rice flour (7–9)
	The smell of cooked glutinous rice flour is not obvious (4–6)
	Unpleasant odor or off odor (1–3)
Hardness	Appropriate strength when chewing (7–9)
	Slightly hard or chewy (4–6)
	Very soft or al dente (1–3)
Graininess	Almost not grainy (7–9)
	Slight grainy (4–6)
	Too grainy (1–3)
Stickiness	Almost not sticky to teeth when chewing (7–9)
	Slightly sticky to teeth when chewing (4–6)
	Too sticky to teeth when chewing (1–3)
Taste	Pleasant taste (7–9)
	Good or normal taste (4–6)
	Poor taste (1–3)
Overall acceptability	Preferable (7–9)
	Normal (4–6)
	Poor (1–3)

**Table A2:** Miller indexes, Bragg angles and interplanar spacings for the orthorhombic phase in rice starch of the glutinous rice flours.

	RF			FRF			HMT-12%			HMT-18%	
*hkl*	2*θ* (°)	*d* (Å)	*hkl*	2*θ* (°)	*d* (Å)	*hkl*	2*θ* (°)	*d* (Å)	*hkl*	2*θ* (°)	*d* (Å)
(020)	9.981	8.855	(200)	15.106	5.8600	(110)	9.041	9.7736	(020)	9.981	8.855
(101)	11.194	7.8981	(031)	17.144	5.1677	(020)	9.981	8.8550	(101)	11.194	7.8981
(200)	15.106	5.8600	(211)	17.959	4.9350	(101)	11.194	7.8981	(200)	15.106	5.8600
(031)	17.144	5.1677	(220)	18.138	4.8868	(200)	15.106	5.8600	(031)	17.144	5.1677
(220)	18.138	4.8868	(141)	23.009	3.8621	(031)	17.144	5.1677	(220)	18.138	4.8868
(310)	23.297	3.8150	(132)	23.684	3.7535	(220)	18.138	4.8868	(141)	23.009	3.8621
(132)	23.684	3.7535				(040)	20.038	4.4275	(312)	28.726	3.1052
(033)	29.251	3.0507				(310)	23.297	3.8150	(060)	30.255	2.9517
(251)	30.630	2.9163				(242)	30.303	2.9471	(400)	30.484	2.9300
(004)	33.503	2.6725				(251)	30.630	2.9163	(440)	36.752	2.4434
(271)	39.676	2.2698				(233)	33.077	2.7059	(262)	38.029	2.3642
						(224)	38.357	2.3448	(510)	38.718	2.3237
	HMT-24%				HMT-30%				HMT-36%		
*hkl*	2*θ* (°)		*d* (Å)	*hkl*	2*θ* (°)		*d* (Å)	*hkl*	2*θ* (°)		*d* (Å)
(020)	9.981		8.855	(011)	9.656		9.152	(020)	9.981		8.8550
(101)	11.194		7.8981	(020)	9.981		8.855	(101)	11.194		7.8981
(200)	15.106		5.8600	(101)	11.194		7.8981	(031)	17.144		5.1677
(031)	17.144		5.1677	(200)	15.106		5.8600	(220)	18.138		4.8868
(220)	18.138		4.8868	(031)	17.144		5.1677	(040)	20.038		4.4275
(040)	20.038		4.4275	(220)	18.138		4.8868	(141)	23.009		3.8621
(141)	23.009		3.8621	(040)	20.038		4.4275	(132)	23.684		3.7535
(051)	26.488		3.3622	(141)	23.009		3.8621	(051)	26.488		3.3622
(242)	30.303		2.9471	(051)	26.488		3.3622	(033)	29.251		3.0507
(251)	30.63		2.9163	(033)	29.251		3.0507	(251)	30.63		2.9163
(161)	32.353		2.7649	(400)	30.484		2.9300	(152)	31.214		2.8631
(004)	33.503		2.6725	(411)	32.048		2.7905	(004)	33.503		2.6725
(303)	34.025		2.6327	(422)	36.380		2.4675	(253)	38.977		2.3089
(224)	38.357		2.3448	(352)	38.175		2.3555				
(510)	38.718		2.3237								

RF: raw glutinous rice flour. FRF: the flour made from stir-fried glutinous rice. HMT-12%, HMT-18%, HMT-24%, HMT-30% and HMT-36% represent the flours made from the glutinous rice subjected to heat–moisture treatment at the moisture content of 12%, 18%, 24%, 30% and 36%, respectively.
